# An Automated System for ECG Arrhythmia Detection Using Machine Learning Techniques

**DOI:** 10.3390/jcm10225450

**Published:** 2021-11-22

**Authors:** Mohamed Sraitih, Younes Jabrane, Amir Hajjam El Hassani

**Affiliations:** 1 MSC Laboratory, Cadi Ayyad University, Marrakech 40000, Morocco; mohamed.sraitih@ced.uca.ma; 2 Nanomedicine Imagery & Therapeutics Laboratory, EA4662—UBFC, UTBM, 90000 Belfort, France; amir.hajjam-el-hassani@utbm.fr

**Keywords:** electrocardiogram, ECG, classification, support vector machines (SVMs), k-nearest neighbors (kNN), Random Forest (RF), voting ensemble, inter-patient paradigm

## Abstract

The new advances in multiple types of devices and machine learning models provide opportunities for practical automatic computer-aided diagnosis (CAD) systems for ECG classification methods to be practicable in an actual clinical environment. This imposes the requirements for the ECG arrhythmia classification methods that are inter-patient. We aim in this paper to design and investigate an automatic classification system using a new comprehensive ECG database inter-patient paradigm separation to improve the minority arrhythmical classes detection without performing any features extraction. We investigated four supervised machine learning models: support vector machine (SVM), k-nearest neighbors (KNN), Random Forest (RF), and the ensemble of these three methods. We test the performance of these techniques in classifying: Normal beat (NOR), Left Bundle Branch Block Beat (LBBB), Right Bundle Branch Block Beat (RBBB), Premature Atrial Contraction (PAC), and Premature Ventricular Contraction (PVC), using inter-patient real ECG records from MIT-DB after segmentation and normalization of the data, and measuring four metrics: accuracy, precision, recall, and f1-score. The experimental results emphasized that with applying no complicated data pre-processing or feature engineering methods, the SVM classifier outperforms the other methods using our proposed inter-patient paradigm, in terms of all metrics used in experiments, achieving an accuracy of 0.83 and in terms of computational cost, which remains a very important factor in implementing classification models for ECG arrhythmia. This method is more realistic in a clinical environment, where varieties of ECG signals are collected from different patients.

## 1. Introduction

**Figure 1:**
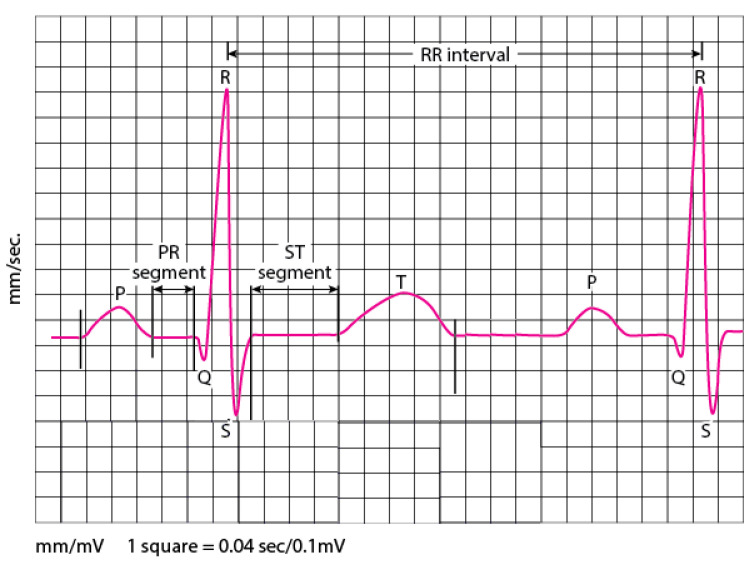
PQRST waves.

One of the most fundamental vital organs is the heart. It’s the engine that pumps blood to many networks of vessels. The heart moves constantly, beating 100,000 times a day by providing oxygen and nutrients while clearing away harmful waste matter. The beating of the heart produces electrical actions measured on the body surface by an electrocardiogram recording (ECG). Skin electrodes record the electrical activity, exposing how each chamber operates in the form of PQRST waves, as illustrated in Figure 1. Therefore, the morphology and heart rate variability (HRV) extracted from the ECG signal reveal the cardiac behavior. The heart behavior analysis expressed by the electrocardiogram signal provides specific information about the heart. Thus, if the ECG is irregular or faster, or slower than normal, that means cardiac arrhythmia. Arrhythmia can cause several types of consequences; an imminent threat to a patient’s life (e.g., ventricular fibrillation and tachycardia), long-term threats, or even more causing death, the thing that made it the most common leading cause of deaths in the world.

Normal cardiac rhythm is occasionally interrupted by a beat that occurs before the regular time of the next sinus beat, and this is described as a premature beat or premature contraction (the terms “ectopic beat” and “extrasystole” are frequently used as synonyms). Originally, the sinus beats start from the SA node, unlike the premature beat, which is preceded by an ectopic focus that may be localized in any section of the heart other than the SA node [1]. Thus, the premature beat is classified into two types depending on the location of the focus; Premature Atrial Contraction (PAC) (also known as an atrial premature beat (APB)) if its origin is above the ventricles, i.e., in the atria or the AV node, or Premature Ventricular Contraction (PVC) (also known as a ventricular premature beat (VPB)) if its origin is in the ventricles. We can recognize the PAC and PVC based on specific characteristics and different circumstances. The usual traditional variety of PAC is linked with an abnormal P wave morphology and a QRS complex morphology matching that of a normal sinus beat, but for the associated compensatory pause; the interval between the two sinus beats that enclose the PAC is less than the length of two normal RR intervals. Unlike the PAC, the presence of a PVC almost always prevents the occurrence of the next sinus beat. Although the SA node discharges on schedule, the impulse cannot propagate to the ventricles because the premature beat has made the tissue refractory. The pause that results between the PVC and the next sinus beat is called the compensatory pause. But the problem with the PVC is that it may originate from any area beyond the point where the common bundle has branched into the left and right bundle branches. And since the electrical impulse of the ventricular ectopic focus does not follow the normal conduction pathways, as a result, the produced QRS complex is abnormally prolonged and has a morphology that deviates considerably from that of a sinus beat (it is often much larger and bizarre-looking). In most cases where the PACs are identified by premature onset and a deformed p wave, it shows the origin of a mostly right atrial “focus” distant from the sinus node [1]. In an aberration of early PACs, the right bundle-branch block is more common than the left bundle-branch block. If the PAC occurs quite early, it may be entirely AV blocked [2]. In these cases, a false diagnosis of the sinuatrial block is sometimes made. If a PAC falls in the probably vulnerable phase of the re-polarization of the preceding atrial beat (“p on Ta”), it may produce atrial fibrillation. Atrial bigeminy is rare; salvos of PACs are rarely observed. In contrast to short episodes of atrial tachycardia, the rhythm is irregular and can lead to diagnostic difficulties. Premature Atrial Contraction (PAC) is less frequent than premature ventricular contractions (PVC) but much more common than super-ventricular premature beats occurring in the atrioventricular (AV) junction. Usually, PACs are not linked with heart disease. However, frequent PACs could be an early symptom of heart failure and may precede atrial fibrillation [3]. More than 80% of deaths in patients with heart failure identify a cause related to cardiovascular disease [4]. In up to 64% of healthy young individuals, some PACs are discovered in an ambulatory electrocardiogram (Holter ECG), and in most samples without symptoms [2]. For the Bundle Branch Block, it occurs when the left/right bundle branch cannot transmit the impulse fast enough. Therefore, the QRS duration is prolonged [3]. Many myocardial diseases are associated with bundle branch block. The prognosis of bundle branch block indicates the underlying myocardial disease and is therefore variable. Mass electrocardiographic surveys show that many apparently healthy individuals have bundle branch block, [5] and in most people, bundle branch block doesn’t cause symptoms. These arrhythmias in the ECG waveform are a sign of cardiovascular complications and are more common in people with underlying heart disease. In terms of diagnosis, it can be detected during a physical examination and confirmed by an electrocardiogram (ECG) recording. But sometimes, doctors must use a Holter monitor to reveal the severity of these arrhythmias, and in some cases, stress tests to perform the right diagnosis and treatment. The hand-operated analysis of ECG signals, especially a Holter recording of three leads, is difficult and time-consuming as it can be a form of 24 h or more of normal daily activities of the patient’s ECG recording. Thus, it requires specialists trained to identify and categorize different waveform morphology in the signal, which remains a hard task, especially for these arrhythmias revealed above, as they are nearly related to each other. A computer-aided diagnosis (CAD) for monitoring cardiac health allows cardiologists diagnostic aid and improves medical decision-making. A large amount of research has been devoted to automated systems for biomedical ECG signal interpretation for early-stage detection of cardiac arrhythmias.

Various types of techniques have been investigated and analyzed for this purpose and other purposes such as decision trees [6], random forest (RF) [7,8], k-nearest neighbors (KNN) [9,10], hidden Markov models [11,12], hyper-box classifiers [13], optimum-path forest [14], conditional random fields [15], besides other methods such as [16,17,18,19,20,21].

Most papers in the literature use the intra-patient paradigm, which is mainly based on beat types. In this way, the ECG recording belonging to the same person can appear in both subgroups (training and testing), and this can lead to a result bias, which makes the classifier produce overly optimistic results [22]. Conforming to the clinical environment, which means real-world clinical practice, the training and test set should contain heartbeats from diverse individuals, and this refers to the inter-patient paradigm. The well-known inter-patient paradigm for ECG beat classification was proposed by [23]. The authors suggested separating the patients into two groups with particular patients, one for training and the other for testing, and the classes employed conformed to the Advancement of Medical Instrumentation (AAMI) standard. They put their method to the test by using extracted features and prior knowledge of ECG morphology; the results were encouraging, but there was still room for improvement. This inter-patient paradigm has been used in several papers [24,25,26,27]. For the non-AAMI standard classes, there are a few papers that worked and used inter-patient paradigms for classes such as Normal Beat (NOR), Left Bundle Branch Block Beat (LBBB), Right Bundle Branch Block Beat (RBBB), Premature Atrial Contraction (PAC), and Premature Ventricular Contraction (PVC) [22,28]. For instance, ref. [28] proposed a combination of three classifiers using the majority vote approach to distinguish three classes, namely Normal, Left Bundle Branch Block Beat (LBBB), and Right Bundle Branch Block Beat (RBBB), and used the inter-patient separation presented by [23]. In their approach, they used a weighted LDA classifier to define the classes NOR and RBBB, a weighted SVM classifier to implement LBBB and NOR, and a minimum distance classifier (MDC) to implement LBBB and NOR. The outcomes were encouraging for each approach, but the results needed improvement when classifying all the classes at once.

However, we believe that there is a better technique to divide patients for an inter-patient paradigm, which would enhance classification results for all classes, particularly those with a small data set. In this work, we introduced a new patient separation approach and compared our results to those of [23].

Several models of machine learning are investigated in the literature for ECG classification. For instance, ref. [29] proposed a classification method using a different set of features, such as empirical and variational mode decomposition and Decision Tree Algorithm, as well as in [8] where the authors extracted different features from the time and frequency domains and used Random Forest as a classifier. These techniques provide promising results, but they cause an interesting computational cost. Authors in [15] suggested the use of weighted Conditional Random Fields for the classification of arrhythmias and compared it with support vector machine (SVM) and LDs. The analysis revealed that the introduced approach gets promising results for the minority arrhythmical classes (SVEB e VEB). In [30] authors used a classifier based on KNN and declared favorable results, though the computational cost was not mentioned. [31] presented a k-nearest neighbor with two types of heartbeat features. [32] presented an SVM classifier with a proposed ‘generalized discrimination analysis based feature selection’ (GDAFS) using an extracted heart rate variability (HRV) from an ECG time series, taking R-to-R periods from each two continuous R points.

**Figure 2:**
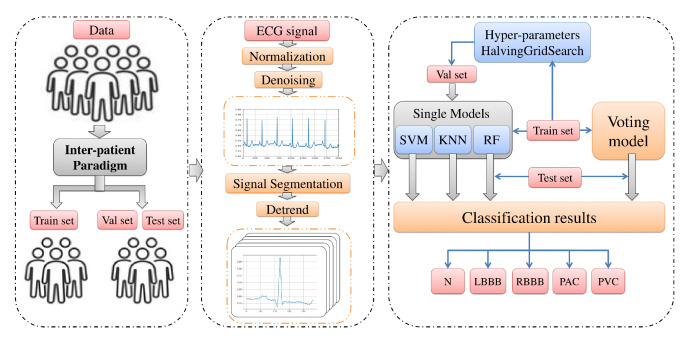
Overall procedures in ECG arrhythmia classification based on proposed models.

In view of the foregoing, we aim in this paper to design and investigate an automatic classification system using a new comprehensive ECG database inter-patient paradigm separation to improve the minority arrhythmical classes detection without performing any feature extraction. We investigated four supervised machine learning models: support vector machine (SVM), k-nearest neighbors (KNN), Random Forest (RF), and the ensemble of these three methods. We test the performance of these techniques in classifying: Normal beat (NOR), Left Bundle Branch Block Beat (LBBB), Right Bundle Branch Block Beat (RBBB), Premature Atrial Contraction (PAC), and Premature Ventricular Contraction (PVC), using inter-patient real ECG records from MIT-DB after segmentation and normalization of the data, and measuring four metrics: accuracy, precision, recall, and f1-score. Figure 2 illustrates a summary of the overall procedures used in this paper.

The rest of this paper is organized as follows: In the next section, we represent the methods. Section 3, describes the results, and a detailed discussion of these results is spread in Section 4. To finish, we conclude the paper in Section 5.

## 2. Machine Learning Models

### 2.1. Support Vector Machines

Support vector machine (SVM) is a well-known classification technique in supervised machine learning [33,34,35]. Practically, the SVM technique performs a classification task by constructing a separating hyper-plane in n-dimensional space (*n* is the number of features used as inputs) that separates different class labels by maximizing the geometric margin between the input data classes mapped in a higher-dimensional space and minimizing the empirical classification error [36,37]. SVM depends in all this classification process on the kernel functions [38], either as a linear or nonlinear classifier according to the type of its kernel function. A linear kernel function makes the SVM a linear classifier. On the other hand, the polynomial and sigmoid kernels make the SVM a non-linear classifier. However, the selection of a good kernel function remains a challenging task. We suppose a training set that consists of N samples ($y_{j}$,$x_{j}$), j=1,…,N, where $x_{j}\in {I\;R}^{n}$ indicates the *n*-dimensional feature vector of the *j*th example and $y_{j}\in I\;R$ signifies the matching class label and $y_{j}\in \pm 1$. A decision function g(x) learned from the training set makes a representation of the optimal hyper-plane that predicts the class label in the subsequent tests. By using the kernel, the decision function is formulated as follow [39,40]:

(1) $$g\left(x\right)=sign\left(\sum_{i\in SV_{s}}\alpha_{i}y_{i}K\left(x,x_{i}\right)+b\right)$$

where α is the Lagrange multiplier for each training data set and $K(x_{j},x)$ is the kernel function that maps the data into higher dimensional space and is defined as in the case of the polynomial Kernel [39]:

(2) $$K\left(x,x_{i}\right)=\sum {\left(x,x_{i}\right)}^{d}$$

where *d* is the degree of the polynomial function.

Essentially, the SVM is a binary classification technique. In order to be extended for a multi-classification task, two techniques are commonly used to make it possible, and these methods are one-versus-one (OVO), one-versus-rest (OVR). In the literature, SVM is one of the most popular classifiers used for other applications in biology [41], specifically for ECG arrhythmia classification [33,42,43,44,45,46].

### 2.2. K-Nearest Neighbors

K-Nearest Neighbors (KNN) is a common supervised machine learning technique and is considered the simplest technique used mostly for classification tasks. KNN is also known as a non-parametric lazy algorithm because it does not use any model to fit, it is only based on memory. Practically, the KNN classifies feature vectors according to the labels of the closest training samples in the feature space. The k-nearest neighbors are collected by calculating the distance (such as Hamming, Euclidean, or Minkowski distance defined in Equation (3)) between an unknown feature vector or new sample and all the vectors in the training set. The unknown feature vector is assigned then to the class to which the closest k samples mostly belong with the help of the votes got from the neighbors [9,47]. The class with the most votes is considered as the prediction. The KNN classifiers require two parameters: the value K and the threshold value. The K value shows the number of nearby neighbors, and the threshold value is used for the evaluation of unusual neighbors.

(3) $$D\left(X,Y\right)={\left(\sum_{i=1}^{n}{\left|x_{i}-y_{i}\right|}^{p}\right)}^{\frac{1}{p}}$$

where *p* is the order and $X=\left(x_{1},x_{2},\ldots ,x_{n}\right),Y=\left(y_{1},y_{2},\ldots y_{n}\right)\in R^{n}$.

The KNN has been widely used and employed in some recent ECG classification studies [20,48,49,50,51].

### 2.3. Random Forest

Random forest (RF) is another famous supervised machine learning technique, which is primarily an ensemble of decision trees to train and predict outcomes, and was proposed first by [52]. The RF is a parametric algorithm regarding the number of trees in the forest and is also a stochastic method because of its two origins of randomness: random attribute sub-set selection and bootstrap data sampling. This randomness helps to avoid over-fitting during the training process. Essentially, the constructed model depends on several parameters. The most important ones are the number of trees, the maximum depth, and the maximum split. The decision trees pick their splitting properties from a random subset of k characteristics at each internal node. The best split is taken within these randomly chosen attributes, and it builds the trees without trimming. RF is universally used in many classification challenges, particularly in areas with larger numbers of attributes and situations, because of its high-speed [7,8,20,53].

### 2.4. Performance Evaluation Measures

There are various metric performance measures to evaluate the classification results. In the literature, four metrics mostly used are [32]:

- *Accuracy*: which can be described as the ratio of exact classification of the total classified outcomes.
  (4) $$Accuracy=\frac{TP+TN}{TP+TN+FP+FN}$$
- *Precision*: also known as positive predictivity, is the ratio of the actual positives within the total number of positively predicted samples and is described as:
  (5) $$Precision=\frac{TP}{TP+FP}$$
- *Recall*: which is also known as sensitivity, is the percentage of positively predicted samples to the total amount of actually positive samples and is defined as:
  (6) $$Recall=\frac{TP}{TP+FN}$$
- *F1-score*: is the consonant mean of precision and recall and is described as:

(7) $$F1-score=2\times \frac{Precision\times Recall}{Precision+Recall}$$

where *TP, TN, FP,* and *FN* are the numbers of true positives, true negatives, false positives, and false negatives, respectively.

All the experiments were run on a laptop equipped with an Intel Core i3 processor and 4 GB of RAM.

## 3. Experimental Results

### 3.1. Data Preparation

To compare the proposed methods in this paper, we used real ECG signals from the MIT-BIH Arrhythmia Database (MITDB) [54]. The MIT-BIH Arrhythmia Database contains 48 half-hours of data. Each record comprises two-channel ambulatory ECG recordings from 47 subjects. The data is digitized at 360 samples per second per channel. The first lead is a changed limb lead II (MLII) for 45 records and a changed lead V5 for the rest. The second lead is the pericardial lead and was conducted as V1 for 40 recordings and V2, V4, or V5 for the others. The original annotation of this data set contains 16 classes of rhythms. The classical method of creating test data is to divide our data into two subsets: the training data set and the test data set. With ECG heartbeat classification, two data separation paradigms are usually used: intra-and inter-patient paradigms. The intra-patient paradigm type divides the data based on the beat type. One of the main weaknesses of this system is that it results in an overoptimistic estimate of the actual classifier performance, because the heartbeats from the same patient can appear in both subsets, the training and test set. The robust split and more realistic to real-world situations is the use of the inter-patient paradigm; here, the records used to train the model are dissimilar to the records used to evaluate it. This ensures that beats from the same patient do not exist in the train and test data sets. It further considers the inter-individual differences and therefore gives non-biased estimates of the classification performance.

### 3.2. New Separation Scheme

In our experiment, we proposed a new separation approach of the patients for an inter-patient paradigm that enhances the classification results for all classes, particularly those with a small data set, which we define as minority. For this purpose, we studied the records and classes in all the records of each patient to ensure the best separation of the patients, taking into consideration that:

- In the inter-patient separation, we must ensure that each patient’s record can only be found in the training or test set. which means each patient’s record is assigned to only one group. In addition, records 201 and 202 belong to the same patient. These two records must be allocated to the same group.
- All the classes have a fair amount of data in each group without affecting the distribution of the patients.
- The five categories, NOR, LBBB, RBBB, PAC, and PVC, should have approximately the same number of samples, as much as possible, in the training and test sets.

In the inter-patient paradigm separation we proposed, the patients are divided into two data groups; each group contains twenty-two records, as the number of patients in each group was proposed in [23], but the separation of the patients is different, it was chosen carefully to maintain the best training of the model on all the features needed for each class to be well detected. We also took into consideration that the data used for the training contains, as possible, more samples of each class than the test set for the model to learn all the features. Both data sets contain normal heartbeats and a mixture of routine and complex arrhythmia recordings. The form of the data separation is described in Table 1.

**Figure 3:**
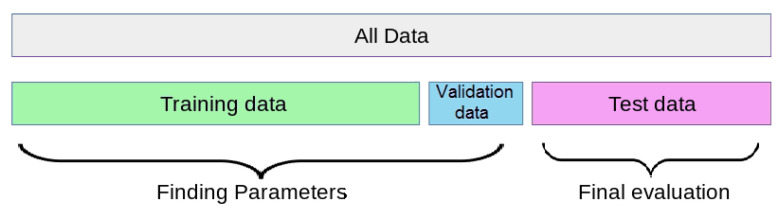
Data separation.

The first data (G1) is considered as the train data set to train the models, it contains data from recordings: [100, 101, 106, 108, 109, 112, 114, 115, 116, 118, 119, 122, 124, 203, 205, 207, 208, 212, 215, 220, 223 and 230]. The second data set (G2) was separated into two sets: 20% for a validation set and 80% for the test set, contain data from recordings: [103, 105, 111, 113, 117,121, 123, 200, 201, 202, 209, 210, 213, 214, 219, 221, 222, 228, 231, 232, 233 and 234]. It is worth noting that Records [102, 104, 107, and 217] are excluded from all the data. We used the validation to validate the model during the experiments, and the test set for the final performance evaluation of the models as described in Figure 3.

### 3.3. Data Pre-Processing

#### 3.3.1. Normalization

We used the first lead of each record from all the data sets and performed normalization of the ECG signal into 0 and 1 using the min-max normalization. This method changes the value of the min limit (a) and max limit (b) on the amplitude of a signal to the desired range with a guarantee of no changing of the pattern or shape of the signal features. In this study, the data was acquired in the pre-process with the min limit (0) and max limit (1), respectively. The mathematical function of the normalization with min-max normalization is as follows:

(8) $$x{}^{\prime \prime }{}^{\prime }=a+\frac{\left(x-x_{min}\right)\left(b-a\right)}{x_{max}-x_{min}}$$

where $x^{\prime \prime }{}^{\prime }$ is the normalized signal, $x_{max}$ and $x_{min}$ are the maximum and minimum values of the data set, respectively, a and b are the min limit and max limit values.

After the normalization process, we denoised the signal using a low-pass Butter-worth digital filter with a cutoff frequency of 0.25 and a filter order of 3 to remove the noise.

#### 3.3.2. Segmentation

**Figure 4:**
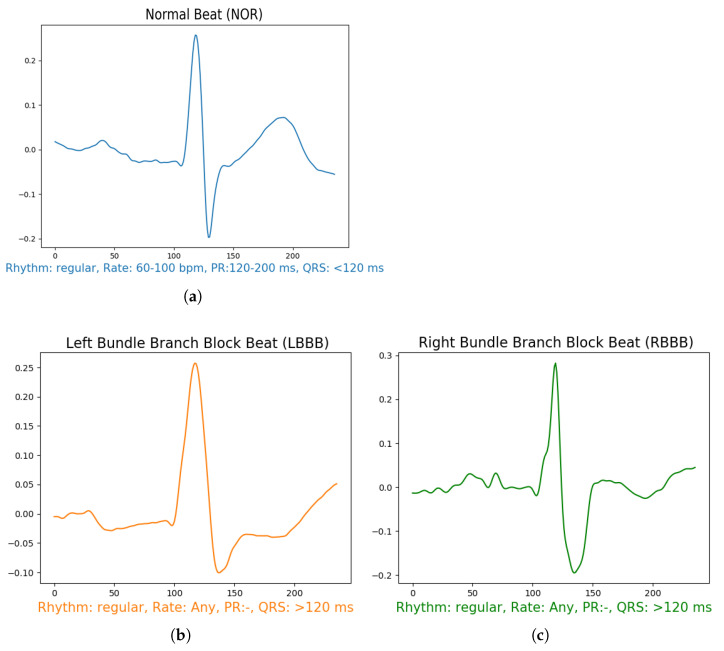

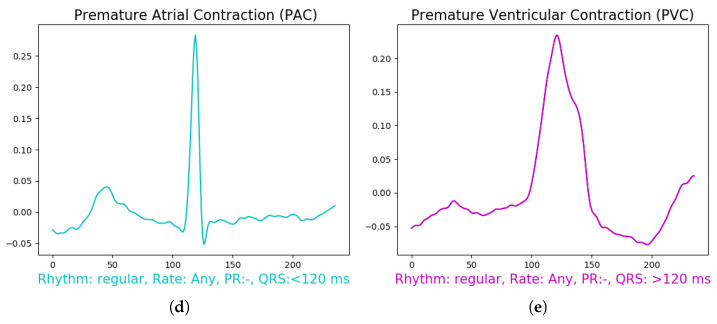
Illustration of different arrhythmias after pre-processing stage including: (**a**) Normal Sinus Rhythm, (**b**) Left Bundle Branch Block, (**c**) Right Bundle Branch Block, (**d**) Premature Atrial Contraction, (**e**) Premature Ventricular Contraction.

After filtering the signal, we performed a sampling of 0.66 s segment for each beat, which means 236 samples in each segment Figure 4, using the annotation data available in the data set for R-peak positions of each beat. The segment’s section is divided into two intervals of t = 0.33 s before and after the annotation position in the signal. From all the data, we used only 5 specific known annotations, from all the labels which are: Normal Beat (NOR), Left Bundle Branch Block Beat (LBBB), Right Bundle Branch Block Beat (RBBB), Premature Atrial Contraction (PAC), and Premature Ventricular Contraction (PVC) as shown in Figure 4. To maintain the fluctuations in the data and yield a better insight of the signal features, we removed a linear trend from the resulting data by calculating the least-squares regression line to estimate the growth rate r. Then subtract the differences (i.e., the deviations from the least-squares fit line) from the data. This method enables the model to focus more on the class features during the training. The final results are demonstrated in Figure 4.

### 3.4. Hyper-Parameters Selection

There are several parameter keys in each model of machine learning to be determined, called Hyper-parameters. The Hyper-parameters help to control the behavior of machine learning algorithms in a way of finding the right balance between bias and variance when optimizing for performance. But there are no fast rules that guarantee the best performance on certain data sets. The classical way to discover the parameters of the model that can achieve the most important prediction results is to perform different varieties of hyper-parameters on each model, and this process is time-consuming. In this paper, we performed a grid search on each model by providing a combination of parameter grids. Table 2 shows the range grid of the hyper-parameters optimized in our experiment.

For the hyper-parameter finding, we used the train set to train the models, and the validation set is considered a test set to validate the models’ performance. The halving grid search algorithm fits the parameters grid on the training set and evaluates its performance on the validation set using successive halving. The successive halving is an iterative selection process where all candidates (the parameter combinations) are evaluated with a few resources at the first iteration. Only some of these candidates are chosen for the next iteration, etc. Thus, only a subset of candidates lasts until the last iteration, which means that is consistently rated among the top-scoring candidates across all iterations. The average of the best-performing model is kept and exposed as the best estimator. All processes are done by using the HalvingGridSearchCV function from the scikit-learn [55]. This experiment outcome revealed the best optimal set hyper-parameters combination that can serve the model in the best way to get the most skillful predictions which will be used for the rest of the experiments in this paper. The hyper-parameter combinations selected are described in Table 3.

These hyper-parameters cannot be taken by blind eyes further to the predictions; they need to be evaluated to indicate the performance of each model over time and to show distinct changes that can accrue and prevent over-fitting and under-fitting of the models.

### 3.5. Qualitative Results

**Figure 5:**
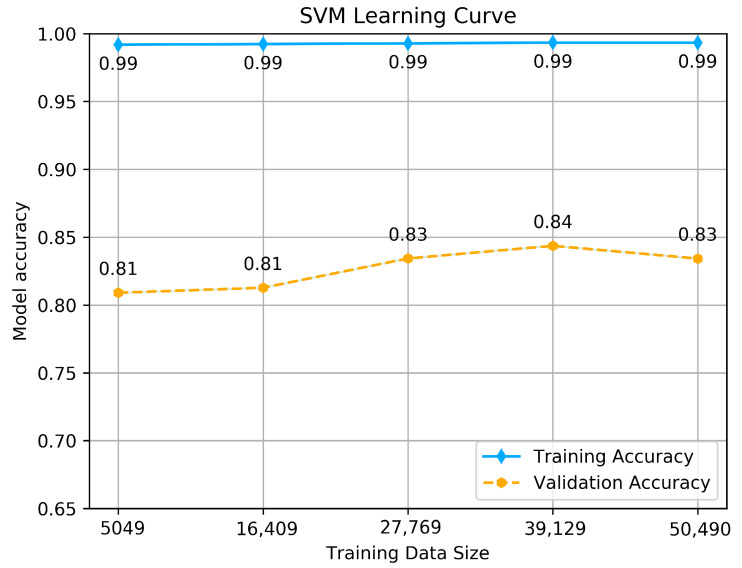
SVM classifier’s Learning curve.

**Figure 6:**
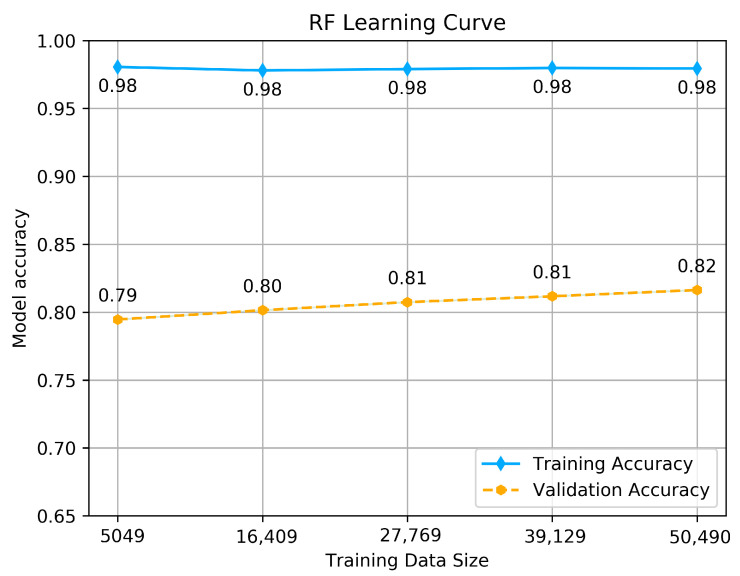
RF classifier’s Learning curve.

**Figure 7:**
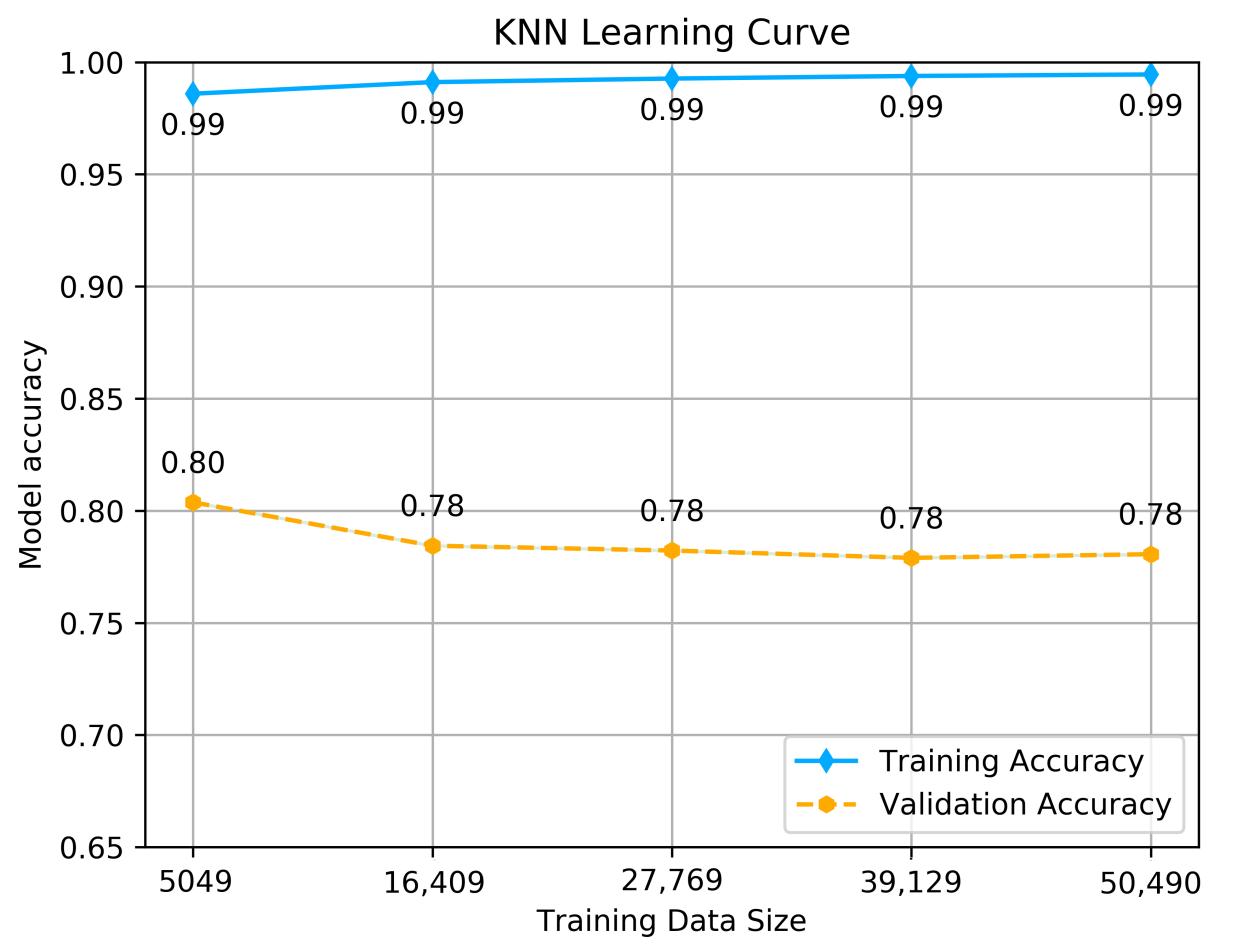
KNN classifier’s Learning curve.

To estimate how the models are expected to perform during training and when used to make predictions on unseen data, to see if the model is well-fitted, we performed a learning curve graph. The learning curve reflects the model’s learning process, which is illustrated by measuring the accuracy of each size of training and validation set that starts from a small data size to the maximum. The accuracy should maximize as more data is fitted, and if it reaches 1 (100% of accuracy), it means the training data set is perfectly learned. The learning curve calculated using a training set shows how the model is learning, and the learning curve calculated from the hold-out validation set provides an idea of if the model is generalizing on unseen data. We tested each model with its hyper-parameters selected above by making a dual learning curve of the training set and a validation set, and measuring the accuracy of each one. The results are shown in Figure 5, Figure 6 and Figure 7.

As we see from the Figure 5, Figure 6 and Figure 7, all the tested models have nearly similar results with few variations. SVM classifier shows almost perfect learning, as we can see from the accuracy achieved by the training set, which goes above 0.99, and it provides promising predictions, as it is obvious from the accuracy achieved using the validation set, which maximizes as more data is fitted, the thing that makes the model learn more features and provide consistent predictions. The maximum accuracy achieved is 0.84. This result reflects that the model is well fitted and generalizes better on unseen data.

The RF classifier shows almost the same behavior as SVM, as we can see from the accuracy achieved by the training set, which goes above 0.98, and as well as provides promising predictions using the validation set, which maximizes linearly as more data is fitted. The maximum accuracy achieved is 0.82. This result reflects that the model is well fitted and generalizes better on unseen data.

Unlike the other classifiers, the KNN model achieves good accuracy on the training set, which goes above 0.99, but it shows different behavior using the validation set. With more data is fitted, the accuracy is exponentially decreasing, with a small variance from 0.80 to 0.78. However, it does not affect the model to be under-fitted or not generalizing on unseen data, and this is just because of the model’s way of dealing with large data, which certainly affects the accuracy with small variance. In the end, we conclude that all the models are well-fitted and well trained to be tested on the test set.

After training the models on the train set and validating them with a validation set, the next step is predicting the outcomes. We tested all the models on the unseen data and the test set. As a performance evaluation, we measured four metrics: accuracy, F1-score, precision, and recall. Table 4 shows the results metrics got from each model in predicting all the classes. For the evaluation comparison of the model’s performance in terms of all metrics, SVM got the best performance results in predicting the outcome of the unseen data by achieving an accuracy of 0.83 and a good precision value of 0.64, followed by RF and KNN classifier, respectively. This performance comparison seems promising to predict all the classes, and this is because the models are well trained on the classes’ features, which can serve better predictions.

**Figure 8:**
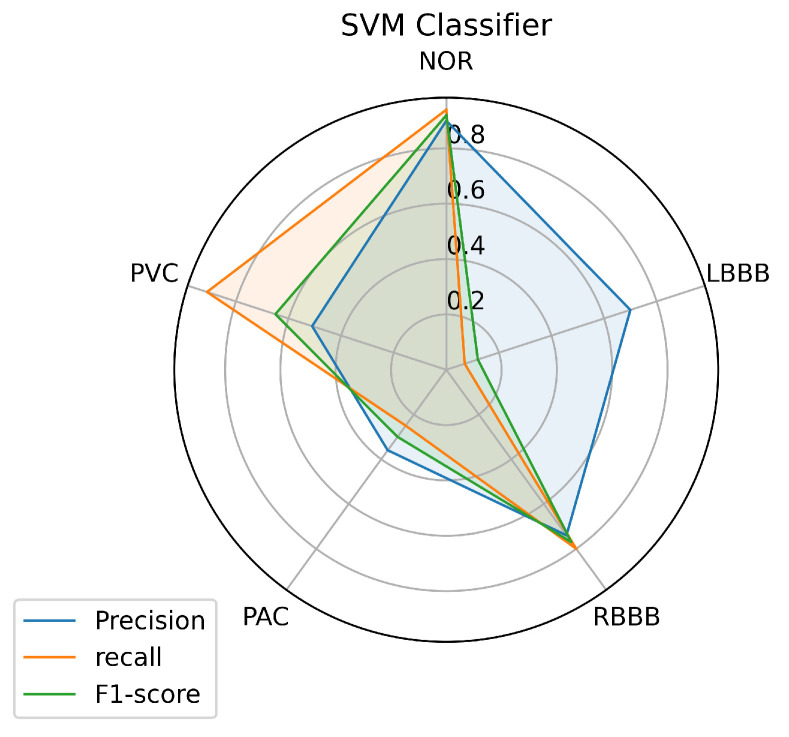
SVM Prediction’s performance on each class.

**Figure 9:**
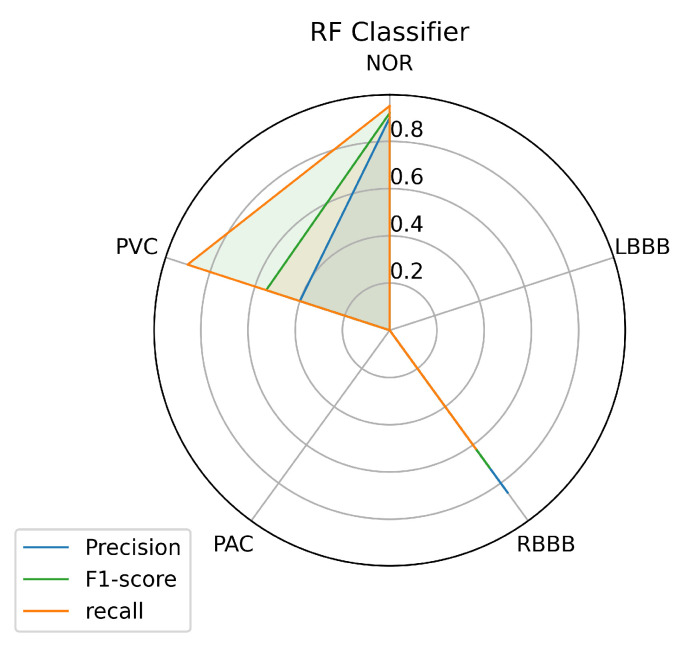
RF Prediction’s performance on each class.

**Figure 10:**
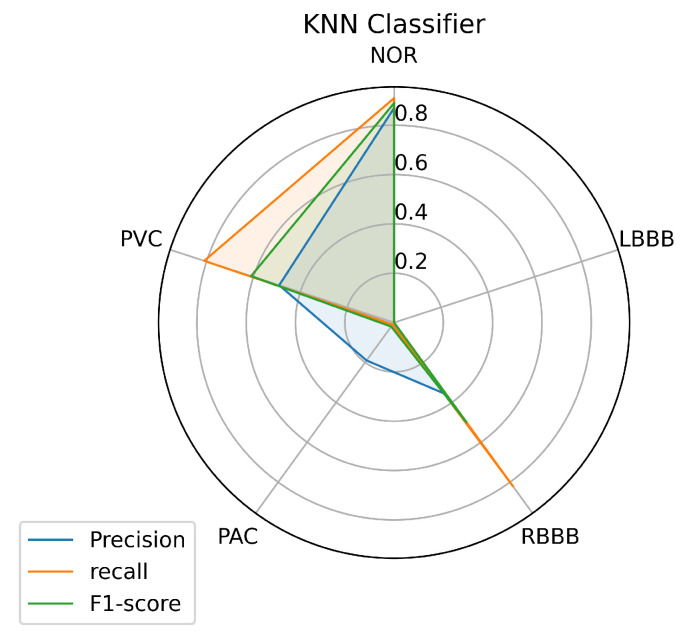
KNN Prediction’s performance on each class.

To have a clear view of the model’s performance on each class, we measured three metrics: precision, recall, and f1-score for each class predicted by the models, and the results are illustrated in Figure 8, Figure 9 and Figure 10.

Figure 8 illustrates the results achieved using the SVM classifier. From the results, it is obvious that the SVM model succeeded in predicting all the classes with different performances. The most predictable classes for SVM are NOR and RBBB, achieving the highest measurement in terms of all metrics which go above 0.75, followed by the PVC. But for LBBB and PAC, it seems to be difficult to predict, as the model didn’t achieve good results that went under 0.50 in terms of all metrics, except for LBBB, which achieved good precision of 0.70. However, it is not surprising for PAC to be less predictable as it has insufficient data in Table 1 for the model to train on because most PACs used in the test set belong to one patient and the rest are from different patients.

Figure 9 and Figure 10 describe the results achieved using the RF and KNN classifiers. The RF and KNN models have the same performance concerning the labels NOR, RBBB, and PVC as the SVM model with little variance. Unlike the SVM, the RF and KNN models didn’t succeed in classifying the LBBB class, even with a sufficient amount of data available for this class for the model to train on. The KNN got a precision of 0.19 in classifying the PAC class, unlike the RF model that didn’t predict it.

We presented the classification report of each model to give an unobstructed view of the number of samples classified into certain classes by each model. Table 5 shows the classification report of the SVM classifier, and it is obvious that the classifier performed well in predicting with a significant percentage of the samples provided as inputs, even for the LBBB and PAC, which remain hard to predict. However, the SVM model has mislabelled some input data, which can be considered a serious issue. As we can see in Table 5, the samples that have an LBBB class, 0.57%, which means half the samples, are predicted as normal cases and 0.35% as PVC classes. The same thing is true for the PAC class, where 0.47% of samples are predicted as Normal (NOR) and 0.21% as RBBB classes. These results cannot be acceptable, at least for the LBBB and the PAC class, as the model didn’t predict more than half of the samples provided. And this is probably because of the weak representation of features in data provided for training the model.

Table 6 shows the classification report of the RF classifier, as we can see from the table, the RF model succeeds to predict just three classes: NOR, RBBB, and PVC, as true classes from the five classes provided with important percentages. The model mislabeled all the other two classes, like other classes. The model classified 0.73% of samples with the class of LBBB as NOR class and 0.25% as PVC class. The same thing goes for the PAC class, where 0.40% of samples are classified as NOR class and 0.53% as PVC class. This behavior is probably because of the model’s way of mapping between inputs and outputs of the data set, which didn’t succeed in recognising the new data provided.

Table 7 describes the classification report of the RF classifier. Unlike the RF classifier, the KNN model predicted four classes: NOR, RBBB, PAC, and PVC as true classes, out of the five classes, with important percentages except for the PAC class, which is considered a very weak percentage, because the model is mistakenly labeling 0.86% of the PAC class as class NOR and the rest as other classes. From the Table 7, the result illustrates that the LBBB class is difficult to be recognized by the KNN model from the data provided, as the model classified 0.63 % of samples as a class NOR and the rest as class PVC.

Although it is clear that the three first classes (NOR, RBBB, and PVC) are the most predictable labels among all the models, this is probably because of the feature clarity of these classes, which makes them easy to learn and predict by the model. The other factor that can help the model learn any class is certainly the amount of data used to train the model for each class, which makes the model learn all the features of a certain label. As a result, it will be easy for the model to figure out the outcomes of each input data set. Sometimes it is difficult for the model to predict nearly perfect some classes even if the data is available, and this problem can occur if the training data doesn’t contain all the features. As we can see, the LBBB label has sufficient training data as detailed in Table 1 but all the models had mislabeling of this class on the unseen data provided. Besides, the poor amount of data can lead to a significant mislabeling as it is obvious for the PAC label, which has a small amount of training data as can be seen in Table 1. However, certain models showed a powerful performance prediction for specific classes; SVM predicted 0.91% of samples for the PVC class. The RF predicted 0.96% of samples for the NOR class, and the KNN model predicted 0.96% of samples for the RBBB class.

To enhance the prediction of certain categories and reduce wrongful predictions of some samples, we investigated the group power of the models tested so far to improve and provide the best predictions, and for this reason, we used a technique called the voting model or majority voting ensemble [56,57] which uses an ensemble of machine learning algorithms. This technique works by combining the predictions from several contribution models and searching for a majority vote for the prediction that will be considered the final prediction of the input. Thus, the voting model style is appropriate when there are two or more models that perform well in predicting certain classes, and that is the case in our experience presented and achieved above. This is to achieve better performance than any single model used in the group. There are two voting methods that the voting model applies, hard voting and soft voting. Hard voting is simply summing up the predictions for each label and taking the prediction that has the most votes. Unlike hard voting, soft voting sums up the predicted probabilities for each class and takes the prediction that has the highest probability. In this work, we used hard voting to keep the performance contribution of each model.

**Figure 11:**
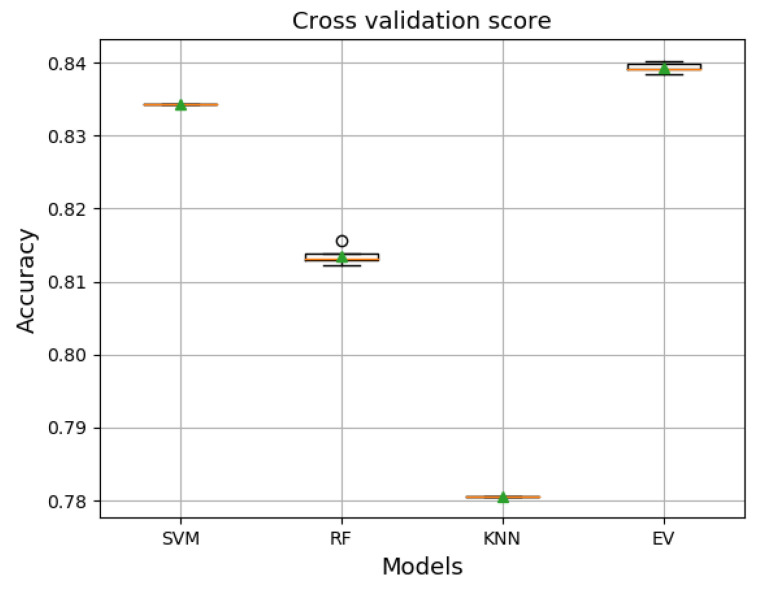
A comparison performance accuracy of the single models and the Voting ensemble.

Figure 11 shows the performance results measured by the cross-validation score technique of each model trained with the training set and validated with the validation set. The results revealed that the ensemble voting achieved the best performance and even better than the other models in terms of average accuracy, as it exceeded an accuracy of 0.83 with a maximum accuracy of 0.84. However, the results are not surprising because the mainly used set of models perform well as a single model, and thus, combining these models with the voting technique would give better performance than the best single model. Concerning the results specified in Figure 11, the ensemble voting method appears to be appropriate and promising to improve the prediction results. Hence, we tested it directly on the unseen data set, the test set.

**Figure 12:**
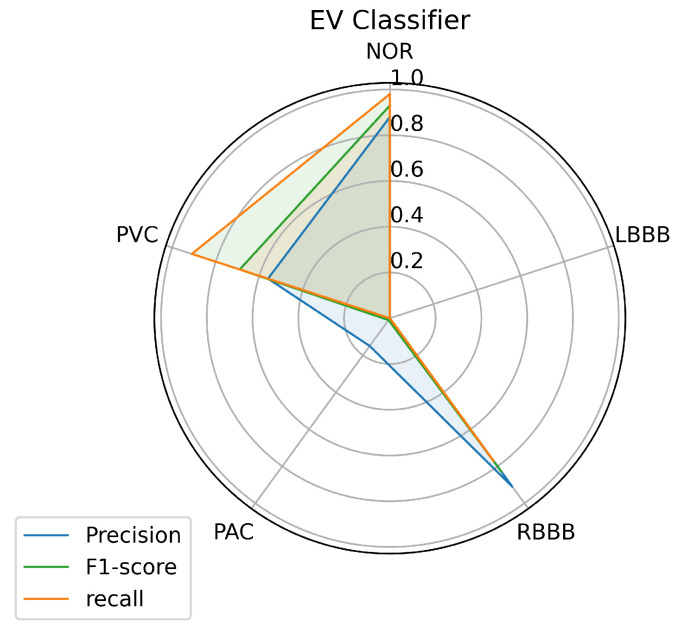
Ensemble voting prediction’s performance on each class.

Figure 12 illustrates the results achieved by measuring the three metrics: accuracy, recall, and f1 score for each class predicted by the ensemble voting classifier using a test data set. From the figure, the ensemble voting model seems similar to the KNN classifier performance. This implies the same performance result with respect to NOR, RBBB, PAC, and PVC labels with little difference. Likewise, for the LBBB class, where the voting model was unsuccessful in its classification. However, the results were surprising, as we expected the model to provide a further improvement, which means better performance than the individual models tested above.

Toward a clear view of comparison, we made a classification report of the predictions on the test data set that shows the results achieved by the voting ensemble. Table 8 shows the classification report for the classifier of the ensemble voting classifier, and it is clear that the classifier did well in predicting samples provided as input, even for PAC, which is difficult to predict. The ensemble voting model predicted 0.98% of the samples provided for the NOR class, which means an improvement of 0.02% above the best single model performance, and this remains a very important improvement. Unfortunately, the model did not improve the prediction of samples for the other classes, as we see from the table. For instance, the prediction of samples for the RBBB class has been reduced by 0.05%, and for the PVC class by 0.01%, and the LBBB class was not predicted by the model.

The results achieved as far by the ensemble voting, in terms of comparison with the results accomplished above, the voting ensemble didn’t get the best prediction results, which was the primary purpose of implementing the voting ensemble. The voting ensemble didn’t perform well, probably because it treats all models the same, which means that all models contribute equally to the prediction. This remains a problem sometimes because some models can be good at some tasks and poor at others, and this doesn’t give the models the chance to provide a powerful performance. This method can affect the results and the improvement of the voting ensemble over the other used models. Otherwise, the voting ensemble has some advantages, such as higher stability or confidence at the expense of the lower mean performance of the ensemble, which may cause the lower variance achieved by the voting ensemble over the other models used in the ensemble.

## 4. Discussion

In real-world practice to analyze the ECG signal, various causes influence the appearance of characteristics in an ECG recording, which include several factors such as gender, age, medical history of the patient and the time of recordings, and previous physical activity, besides the wide variability in ECG morphological and temporal features within a population. This makes medical decision-making a challenging task that requires more tests and even specialists to deal with it. There are confusing factors where unusual beat morphologies may be recognized for the same disease or similar ones for different diseases, e.g., the PAC and PVC, etc. [2]. For this kind of situation, the cardiologists need to process vast amounts of data of extended ECG recordings of several hours to observe the status of patients’ health. Furthermore, making other tests like the Holter ECG or stress tests that require a significant amount of time, [2]. Such scenarios bring the need for an automated arrhythmia detection system that offers diagnostic aid to cardiologists to improve medical decision-making in a limited time.

An automated arrhythmia detection system needs to be reliable, with high quality and performance in decision making, with less complexity, especially concerning the number of resources required to run it, and more realistic in a clinical environment. Considering these requirements and the metrics used in this paper, the results achieved with all the machine learning models we investigated in this paper, and using our proposed inter-patient paradigm separation method of the patient concerning the MIT-BIH DB, and applying no complicated data pre-processing or feature engineering methods, prove that the SVM is the model that outperformed the other techniques, in predicting the most classes presented such as (NOR, RBBB, and PVC), and especially the minority ones such as the PAC, as it predicted one quarter of the data provided, which was the purpose of the inter-patient paradigm separation proposed. Over all models, it is noticeable that the three individual classes (NOR, RBBB, and PVC) are the most predictable labels, as we can observe from the Figure 8, Figure 9 and Figure 10. This is most likely due to the feature clarification of these classes, which makes them easier for the model to learn and predict. Another aspect that can assist the model in learning any class is the amount of data utilized to train the model for each class, which allows the model to learn all or most of the characteristics of a specific label. Thus, it increases the chances of the best predictions. However, this is not necessary and the proof for that is the LBBB class, as it has a fairly sufficient amount of training data, in comparison with other classes investigated in Table 1, but all the models miss-predicted and didn’t recognize it among the data provided, except for the SVM model which at least predicted some amount. And this is probably because the data doesn’t contain all the features needed for this class to be identified on unseen data. However, certain models showed powerful performance predictions for specific classes, such as SVM for the PVC class, RF for the NOR class, and the KNN model for the RBBB class. This phenomenon is caused by the model’s methodology of mapping between the data set’s outputs and inputs, which drives the model to strongly identify particular classes over others. In the case of SVM, the polynomial kernel chosen by the HalvingGridsearch technique, which allows for curved lines in the input space beside a more flexible decision boundary [58]. Despite the powerful performance on specific classes of each model, the ensemble voting model didn’t much succeed in enhancing the predictions performance by using all the models. Instead, it decreased the performance prediction of some classes, except for class NOR, which decreased by 0.02% and failed to predict other classes. This is due to the fact that ensemble voting considers all models evenly, which means that all models vote equally for the predictions. This remains an obstacle to the minority classes, as it is predicted by a few models, and makes the majority models votes less, or against, this class as they do not recognize it and limit its chance of being predicted. as a result, increasing the performance of the majority of the predicted classes at the expense of the minority predicted classes. Thus, single model performance is preferred in such scenarios, which is in our case, the SVM model. And it can be considered a more realistic and generic approach for dealing with scenarios in which a variety of ECG signals are collected from different patients, whose ECG data was not available during the training phase, as it is trained with an inter-patient paradigm data. On another hand, the SVM model has a low and stable computational cost in terms of time during training and predicting the outcomes. The other models have a high computational cost, in terms of time, either at the training or prediction stages, as we can see, for illustration purposes, in Table 9.

The data provided remains very important to train the model on classes and provides an unrestricted view of features of each class, which makes the prediction more feasible. For the inter-patient paradigm separation, where the patients are divided into two data groups, we chose carefully the data used for the training in such way it contains, as possible, more samples of each class than the test set to ensure the best training of the model on all the features. The proposed separation inter-patient paradigm of the patients used in this paper made a significant improvement in predicting samples of certain classes, e.g., the RBBB class, and especially improved the minority arrhythmical classes detection without performing any features engineering complexity, e.g., the PAC class, which remains challenging to predict by most models.

**Figure 13:**
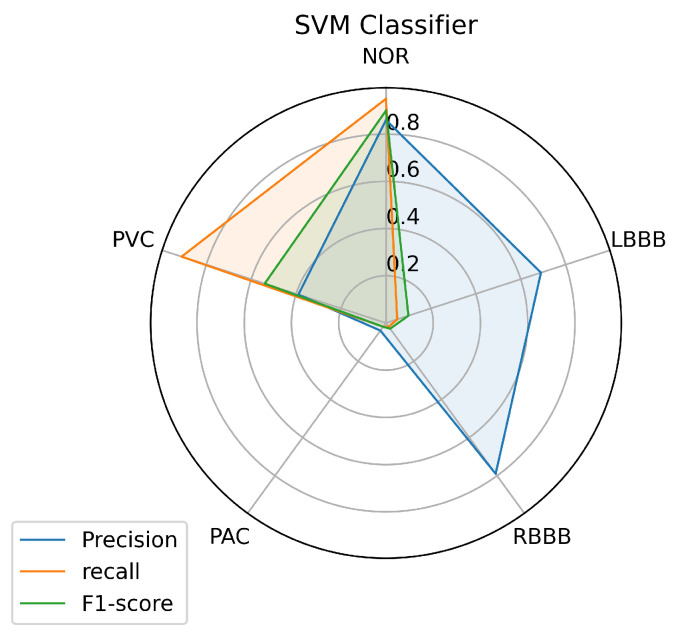
SVM Prediction’s performance on each class using the inter-patient paradigm separation of [23].

For a fair comparison, we used the outperformed model, the SVM model in our proposed separation approach, and evaluated the inter-patient paradigm proposed by [23], using the same hyper-parameters used above. We measured three metrics: accuracy, recall, and f1 score for each class predicted by the SVM model using the [23] separation approach. The results are shown in Figure 13. In comparison with the results described in Figure 8, our separation approach appears to be more effective and promising for all classes, including LBBB, and especially for the minority class PAC.

Likewise, the results are supported by the classification report in Table 10 in comparison with the results in Table 5.

As there has been no previous inter-patient research for the classification of specific types of arrhythmia, specifically the kind we used in terms of an inter-patient paradigm, we also compared the performance of the proposed scheme with other studies in the literature, with one similar work. The overall accuracy achieved by the proposed method seems better than similar work, which uses feature extraction and nearly the same models with different inter-patient schemes and parameters. Besides better results in terms of sensitivity and positive predictivity, considering the same model, the SVM model, and competitive ones considering their performed model. The result we found compared to other works that were conducted following the AAMI standard, which means a superclass of arrhythmia as shown in Table 11, demonstrates that our proposed structure appears to compete with other papers that use the inter-patient [23] paradigm. However, it is worth noting that a superclass, e.g., N, contains NOR, LBBB, and RBBB classes, etc., and in comparison to superclasses of arrhythmia, specific types of arrhythmia are difficult to classify. This gives the proposed work the potential to be very competitive to perform well, and shows that our proposed inter-patient model could provide even better performance if used with feature extraction, which will compete with the state-of-the-art, the results are shown in Table 11.

## 5. Conclusions

In this paper, we investigated an automatic classification system using a new comprehensive ECG database inter-patient paradigm separation to improve the minority arrhythmical classes detection without performing any features extraction. We tested four supervised machine learning models: support vector machine (SVM), k-nearest neighbors (KNN), Random Forest (RF), and the ensemble of these three methods. We tested the performance of these techniques in classifying: Normal Beat (NOR), Left Bundle Branch Block Beat (LBBB), Right Bundle Branch Block Beat (RBBB), Premature Atrial Contraction (PAC), and Premature Ventricular Contraction (PVC), using inter-patient real ECG records from MIT-DB after segmentation and normalization of the data, and measuring the accuracy, precision, recall, and f1-score. The simulation shows that the SVM had the best performance in comparison with the other methods using the proposed inter-patient paradigm separation, which reflects the importance of this method as a classification tool for ECG arrhythmia.

## Figures and Tables

**Table 1:** Heartbeat types with the number of samples of, group 1 (G1) and group 2 (G2) from the MIT-BIH.

Data Set	Heart Beat Type	NOR	LBBB	RBBB	PAC	PVC
G1	Number of total records	37,018	3949	5608	430	3485
G2	Number of total records	37,528	4126	1651	2116	3418

**Table 2:** Optimized Hyper-parameters for each model.

Models	Range of Grid
SVM	kernel = [‘linear’, ‘poly’, ‘rbf’, ‘sigmoid’], C = [ 10, 100, 1000], degree = [2, 3, 4]
kNN	metric = [‘euclidean’, ‘manhattan’, ‘minkowski’], n-neighbors = [1:21]interval:2, weights = [‘uniform’, ‘distance’]
RF	n-estimators = [100, 200, 400, 800, 1000], criterion = [‘gini’, ‘entropy’], max-depth = [5, 15, 25], min-samples-split = [5, 10, 15, 100]

**Table 3:** Selected hyper-parameters.

Models	Final Hyper-Parameter Values
SVM	C = 10, degree = 2, kernel = ‘poly’
kNN	metric = ‘minkowski’, n-neighbors = 11, weights = ‘uniform’
RF	n-estimators = 800, max-depth = 5, min-samples split = 15

**Table 4:** Prediction’s performance comparison.

Classifier	Accuracy	F1-Score	Precision	Recall
SVM	**0.83**	**0.55**	**0.64**	**0.59**
RF	0.82	0.43	0.42	0.49
KNN	0.78	0.40	0.38	0.50

**Table 5:** SVM classification report.

		Predicted Label				
		NOR	LBBB	RBBB	PAC	PVC
True Label	NOR	**0.94**	0.0019	0.00033	0.023	0.034
	LBBB	0.57	**0.068**	0	0.013	0.35
	RBBB	0.091	0	**0.80**	0.023	0.089
	PAC	0.47	0.01	0.21	**0.25**	0.065
	PVC	0.07	0.0077	0.0055	0.004	**0.91**

**Table 6:** RF classification report.

		Predicted Label				
		NOR	LBBB	RBBB	PAC	PVC
True Label	NOR	**0.96**	0.0023	0.00063	$3.3\times 10^{-5}$	0.039
	LBBB	0.73	**0**	0.00061	0	0.27
	RBBB	0.0068	0	**0.61**	0	0.38
	PAC	0.4	0	0.074	**0**	0.53
	PVC	0.095	0.0018	0.0066	0.00074	**0.90**

**Table 7:** Classification report: KNN.

		Predicted Label				
		NOR	LBBB	RBBB	PAC	PVC
True Label	NOR	**0.91**	0.00013	0.06	0.0023	0.03
	LBBB	0.63	**0**	0	0	0.37
	RBBB	0.14	0	**0.82**	0	0.039
	PAC	0.86	0	0.037	**0.012**	0.087
	PVC	0.11	0.063	0.0052	0.0081	**0.81**

**Table 8:** Ensemble voting classification report.

		Predicted Label				
		NOR	LBBB	RBBB	PAC	PVC
True Label	NOR	**0.98**	0	0.00037	0.0009	0.021
	LBBB	0.65	**0**	0	0	0.35
	RBBB	0.15	0	**0.77**	0	0.087
	PAC	0.89	0.00059	0.056	**0.0035**	0.051
	PVC	0.089	0.0029	0.0015	0.0026	**0.90**

**Table 9:** Computational cost comparison.

Methods		SVM	RF	KNN	VE
Cost	Training	39.58 s	478.64 s	0.031s	508.05 s
	Prediction	19.99 s	7.61 s	135.74 s	159.85 s

**Table 10:** SVM classifier, with the same hyper-parameters used above, classification report using [23] inter-patient paradigm.

		Predicted Label				
		NOR	LBBB	RBBB	PAC	PVC
True Label	NOR	**0.95**	0.0015	0.00027	0.016	0.035
	LBBB	0.62	**0.052**	0	0.018	0.31
	RBBB	0.44	0	**0.018**	0.0011	0.54
	PAC	0.84	0.0093	0	**0.016**	0.13
	PVC	0.076	0.0078	0.0019	0.005	**0.91**

**Table 11:** Comparison with other works in the literature.

Ref.	FeEx	CM	Acc(%)	Se	${\mathrm{Pe}}^{+}$	Se	${\mathrm{Pe}}^{+}$	Se	${\mathrm{Pe}}^{+}$	Se	${\mathrm{Pe}}^{+}$	Se	${\mathrm{Pe}}^{+}$
				N		S		V		F		Q	
De Chazal et al. [23]	Yes	LD	85.8	86.8	99.1	75.9	38.5	77.7	81.9	89.4	8.6	0.0	0.0
Chen et al. [59]	Yes	SVM	93.1	98.4	95.4	29.5	38.4	70.8	85.1	0.0	0.0	0.0	0.0
				**NOR**		**LBBB**		**RBBB**		**PAC**		**PVC**	
Shi et al. [22]	Yes	SVM	60.0	98.0	84.6	0.5	11.9	36.7	87.6	76.4	34.6	88.6	75.7
		EV*	74.5	95.0	85.1	27.9	83.8	79.6	88.9	81.8	54.6	88.1	76.0
Proposed Method	No	SVM	**83.0**	94.0	**90.0**	0.7	70.0	**80.0**	74.0	25.0	36.0	**91.0**	51.0

EV* is an ensemble voting of four models, KNN, SVM, DT, and RF.

## Data Availability

Not applicable.

## Abbreviations

We have listed all the technical acronyms used in this paper:

ECG	Electrocardiogram
SVM	Support vector machine
RF	Random forest
KNN	K-Nearest Neighbors
EV	Ensemble voting
LD	Linear Discriminant
NOR	Normal beat
LBBB	Left Bundle Branch Block Beat
RBBB	Right Bundle Branch Block Beat
PAC	Atrial Premature Contraction
PVC	Premature Ventricular Contraction
AAMI	Association for the Advancement of Medical Instrumentation
FeEx	Feature extraction
CM	Classifier Model
Se	Sensitivity
$Pe^{+}$	Positive predictivity
N	Nonectopic beat
S	Supraventricular ectopic beat
V	Ventricular ectopic beat
F	Fusion beat
Q	Unknown beat
Ref	reference
